# Adherence of North-African Pulmonologists to the 2017-Global Initiative for Chronic Obstructive Lung Disease (GOLD) Pharmacological Treatment Guidelines (PTGs) of Stable Chronic Obstructive Pulmonary Disease (COPD)

**DOI:** 10.1155/2020/1031845

**Published:** 2020-02-28

**Authors:** Sana Aissa, Asma Knaz, Jihene Maatoug, Ahmed Khedher, Wafa Benzarti, Ahmed Abdelghani, Abdelhamid Garrouche, Abdelaziz Hayouni, Mohamed Benzarti, Imen Gargouri, Helmi Ben Saad

**Affiliations:** ^1^Department of Pneumology, Farhat Hached University Hospital in Sousse, Tunisia; ^2^Interaction of the Cardiorespiratory System (LR14ES05) Research Laboratory, Faculty of Medicine of Sousse, University of Sousse, Tunisia; ^3^Université de Sousse, Faculté de Médecine de Sousse, Département d'épidémiologie, Hôpital Farhat Hached, Sousse, Tunisia; ^4^Medical Intensive Care Unit, Farhat Hached University Hospital in Sousse, Tunisia; ^5^Physiology Laboratory, Faculty of Medicine of Sousse, University of Sousse, Tunisia; ^6^Department of Physiology and Functional Exploration, Farhat Hached University Hospital in Sousse, Tunisia; ^7^Heart Failure (LR12SP09) Research Laboratory, Farhat Hached University Hospital in Sousse, Tunisia

## Abstract

**Background:**

No previous study has investigated the adherence rate of North-African pulmonologists to the 2017-GOLD PTGs.

**Aims:**

To investigate the adherence rate of Tunisian pulmonologists to the 2017-GOLD PTGs and to identify the barriers to their adherence.

**Methods:**

This was a cohort study involving clinically stable COPD patients who presented to a pulmonology outpatient consultation. The patients were classified as having been appropriately and inappropriately (over- or undertreatment) treated for the GOLD group. Logistic regression was performed to determine the adherence barriers to the 2017-GOLD PTGs.

**Results:**

A total of 296 patients were included (88.1% males, mean age: 68 ± 10 years; GOLD A (7.1%), B (36.1%), C (4.1%), and D (52.7%)). The pulmonologists' adherence rate to the 2017-GOLD PTGs was 29.7%. There was a significant statistical difference between the adherence rates among the four GOLD groups (A: 19.0%, B: 20.6%, C: 8.3%, and D: 39.1%; *p* = 0.001). Differences were statistically significant between the GOLD group D and groups B (*p* = 0.001). Differences were statistically significant between the GOLD group D and groups B (*p* = 0.001). Differences were statistically significant between the GOLD group D and groups B (

**Conclusion:**

The adherence rate of Tunisian pulmonologists to the 2017-GOLD PTGs is low. It seems that the patients' age, socioeconomic level, national health insurance coverage, and GOLD groups influenced their adherence.

## 1. Introduction

The 2017-Global Initiative for Chronic Obstructive Lung Disease (GOLD) guideline, published online in December 2016, presented new recommendations regarding chronic obstructive pulmonary disease (COPD) pharmacologic and nonpharmacologic treatments [1]. It also provided suggestions with regard to the way pulmonologists can integrate these recommendations into COPD care practices [1, 2]. However, although pulmonologists are the frontline healthcare professionals throughout the COPD continuum of care, their understanding of some GOLD pharmacological treatment guidelines (PTGs) is questioned [3, 4]. First, a large inequality of pulmonologists' adherence rates to the GOLD PTGs was reported worldwide (e.g., rates varied from 26.3 (Greece [5]) to 61.4% (Italy [6]) in Europe, from 18.7% [7] to 40% [8] in the USA, and from 44.9% (Taiwan [9]) to 49.6% (South Korea [10]) in Asia, and a low adherence rate was reported in Latin-America (36.3% in Brazil [11])). Second, several barriers were advanced to explain the aforementioned variability in the adherence rates to the GOLD PTGs [1, 12, 13]. According to the World Health Organization (WHO) [14], these barriers are frequently related to different aspects of the problem (e.g., social and economic factors, healthcare team/system, characteristics of the disease, disease therapies, and patient-related factors). In case of the COPD, the barriers can be related to the physician (e.g., knowledge, nonagreement, or nonfamiliarity with the recommendations, lack of awareness and/or of educational material/support, time constraints, and outcome of expectancy), patient (e.g., understanding, age, sex, race, smoking status, schooling level, socioeconomic level (SEL), length of COPD diagnosis, and number of concomitant treatments), guideline (e.g., presence and number of exacerbations, COPD assessment test (CAT) score, and disease severity), and/or social (e.g., treatment costs or availability, environmental factors) [5, 12–23].

In Africa, the data related to the pulmonologists' understanding of the 2017-GOLD PTGs (pulmonologists' adherence rates and/or barriers) are lacking. For example, in North-Africa (Tunisia, Algeria, Morocco, Libya, Mauritania, and Egypt), despite the fact that COPD is frequent [24], health authorities had no information regarding the pulmonologists' adherence rate and/or barriers to the 2017-GOLD PTGs [1]. To the best of the authors' knowledge, no previous North-African study has investigated the aforementioned issue. This crucial situation presents a handicap for any future planning action. In fact, pulmonologists' adherence to appropriate therapies is a major determinant of the treatment success. A low adherence reduces the optimum clinical benefits and therefore attenuates the overall efficiency of any health system [14].

According to the WHO [14], “additional research is needed on the rates of adherence and barriers to adherence in developing countries.” Taking into account the abovementioned points, the main aim of this study was to determine the adherence rate of Tunisian pulmonologists to the 2017-GOLD PTGs. The second aim was to identify their adherence barriers. Tunisian pulmonologists managing COPD are supposed to perfectly adhere to the 2017-GOLD PTGs.

## 2. Population and Methods

### 2.1. Study Design

This was a cohort study conducted from January to December 2017. It was carried out in the Pulmonology Department at Farhat Hached University Hospital (Sousse, Tunisia).

### 2.2. Population

The population source was formed by COPD patients presenting to the outpatient consultation of the abovementioned pulmonology department. The target population was formed by COPD patients who consulted during the study period. Only the patients with a confirmed COPD diagnosis and aged ≥40 years at the moment of enrollment in the study were included. The patients with known concomitant chronic pulmonary diseases (e.g., asthma, lung cancer, and pulmonary fibrosis) or presenting an acute COPD exacerbation were not included.

### 2.3. Sample Size

The sample size was estimated using a predictive formula [25], detailed in [Supplementary-material supplementary-material-1] . The sample size for the study was 298 COPD patients.

### 2.4. Data Collection

COPD patients' data were collected from their sheets/records stored in the outpatient consultation archive. The pulmonology department, including ten pulmonologists (six professors, two assistants, and four residents), has an electronic database of all the examined patients. The patients' records are encoded using specific keywords. In this study, the applied keyword was “COPD.” Each patient's sheet/record includes the following information: sociodemographic (i.e., sex, birth date, location of residence, SEL, and health insurance status), clinical (i.e., smoking habits, comorbidities), and COPD (i.e., spirometric data, dyspnea, GOLD stage/group, exacerbation history, and pharmacologic and nonpharmacologic treatments) characteristics. Incomplete coded sheets/records were excluded.

The following three sociodemographic data were noted: location of residence (urban/rural), SEL (low (unemployed), medium (manual employees), and high (skilled and professional employees)) [26], and health insurance status (indigent, national health insurance, and no insurance).

Cigarette smokers were divided into three groups [27]: nonsmokers, former smokers (smokers who had stopped smoking for more than one year), and current smokers (people who still smoke). Heavy smokers were patients who smoked more than 20 pack-years. Two groups of patients were identified according to whether they were exposed to biomass or not [28]. The patient's comorbidities were noted.

The following COPD characteristics were noted: (i) postbronchodilator (PBD) forced expiratory volume in one second (FEV_1_, L, %), forced vital capacity (FVC, L, %), FEV_1_/FVC ratio (absolute value); (ii) GOLD stage; (iii) modified British Medical Research Council (mMRC) scale for dyspnea (detailed in [Supplementary-material supplementary-material-1]) [29]; (iv) exacerbation risk; and (v) GOLD group. Spirometric and bronchodilator tests were performed according to the international guidelines [30]. Local spirometric norms were used [31]. COPD exacerbation was defined as an acute event characterized by a deterioration of the patient's respiratory symptoms that are beyond the normal day-to-day variations and which leads to treatment modification [32].

The following pharmacologic treatments were noted: short-acting bronchodilators (*β*_2_-agonists (SABA), muscarinic agonists (SAMA)), long-acting bronchodilators (*β*_2_-agonists (LABA), muscarinic antagonists (LAMA)), inhaled corticosteroids (ICS), and theophylline. Only earlier long-term treatments used by stable COPD patients at the time of their inclusion in the study were considered. Long-term treatments were those prescribed by specialists at the time of inclusion in the database and which were extended several months later. Nonpharmacologic treatments included smoking cessation, influenza vaccination, pulmonary rehabilitation, long-term oxygen, and noninvasive ventilation.

### 2.5. Applied Definitions and Classifications

COPD diagnosis was retained when the PBD FEV_1_/FVC *ratio* < 0.70 [1]. Four GOLD stages (mild, moderate, severe, and very severe), detailed in [Supplementary-material supplementary-material-1], were identified [1]. The refined “ABCD” assessment tool derived exclusively from the patient's symptoms (dyspnea, mMRC) and the exacerbation history (including prior hospitalizations) was applied [1]. Four GOLD groups (A, B, C, and D) were categorized [1]. The patients who developed two or more exacerbations per year were qualified as frequent exacerbators [33].

Pulmonologists' adherence to the guidelines was defined as “conformity in fulfilling or following official, recognized, or institutional requirements, guidelines, recommendations, protocols, pathways, or other standards” [34]. The appropriateness of the pharmacologic treatment and the type of inappropriateness were established in accordance with the 2017-GOLD PTGs [1]. Appropriate treatment was defined as using the first drug choice or the alternative choice recommended in the 2017-GOLD PTGs [1]. Inappropriate therapy was classified as over- or undertreatment in accordance with the categorization in the GOLD guidelines. In clinically stable COPD patients, the 2017-GOLD PTGs are dependent on the GOLD groups (Table 1) [1].

### 2.6. Statistical Analysis

The Kolmogorov-Smirnov test was used to analyze the distribution of variables. When the distribution was normal and the variances were equal, the results were expressed by their *mean* ± *standard* *deviation* and 95% confidence interval. When the distribution was not normal, the results were expressed by their median (interquartile range). Qualitative data were expressed as relative number (%). The categorical variables were analyzed using a chi-square test for independent samples. Comparisons of the percentages of patients adhering to the 2017-GOLD PTGs across the GOLD groups were accomplished via the Pearson chi-square test. When applicable, significant differences between the percentages were tested using the McNemar test. The factorial analysis of variance was used to compare the quantitative variables. It was then followed by the Tukey test. The odds ratio (OR) was calculated using the logistic regression test during the uni- and multivariate analysis. Only identified significant factors during the univariate analysis were included in the multivariable one. The level of statistical significance was set at 0.05. All statistical analyses were performed using SPSS 20.0 software.

## 3. Results

### 3.1. General Characteristics

Among the 350 medical records, 54 were excluded mainly because of missing data.  Table 2 displays the general characteristics of the 296 COPD patients.

### 3.2. Tunisian Pulmonologists' Adherence Rates to the 2017-GOLD PTGs

**Figure 1:**
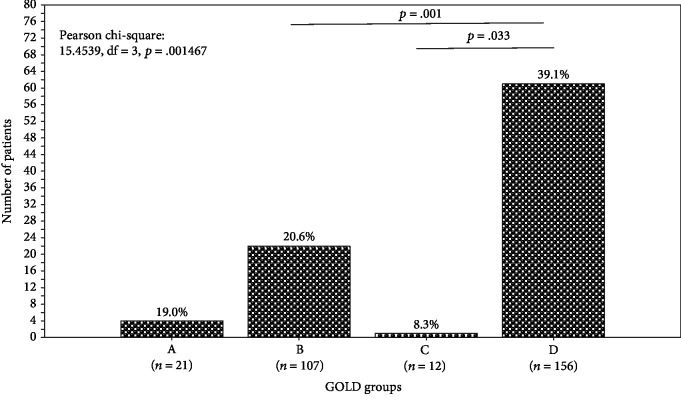
Number (%) of patients having an appropriate medical treatment according to the 2017-GOLD guidelines across the four ABCD GOLD groups. GOLD: Global Initiative for Chronic Obstructive Lung Disease.

There was a significant difference between the pulmonologists' adherence rates to the 2017-GOLD PTGs across the GOLD groups (Figure 1). The difference was significant between the group D (39.1%) and the groups B (20.6) and C (8.3%). The overall adherence rate was 29.7% (*n* = 88).

For each GOLD group, there was a significant difference between the percentages of patients with appropriate and inappropriate pharmacologic treatments (Table 3). The most inappropriate pharmacologic treatments were the associations SABA-ICS in groups A (33.3%) and B (29.9%), SABA-LABA-ICS-Theophylline in group C (33.3%), and SABA-ICS-Theophylline (21.1%) as well as SABA-ICS (19.9%) in group D.

### 3.3. Adherence Barriers to the 2017-GOLD PTGs

Twelve factors influenced the Tunisian pulmonologists' adherence to the 2017-GOLD PTGs (Table 4): sex, age (absolute data and younger group), SEL, national health insurance coverage, smoking habits (current or ex-smoker and exposition to biomass smoke), follow-up by a pulmonologist, frequent exacerbator, and GOLD groups. The multivariate analysis retained only the following four factors: age, SEL, national health insurance coverage, and GOLD groups (Table 5).

## 4. Discussion

The main finding of this study involving 296 COPD patients was a low adherence rate (29.3%) of Tunisian pulmonologists to the 2017-GOLD PTGs. It seems that the patients' age, SEL, national health insurance coverage, and GOLD groups influenced the Tunisian pulmonologists' adherence to the 2017-GOLD PTGs.

In developing countries, such as the North-African ones, when combined with a meagre access to healthcare, a lack of correct diagnosis, and a restricted access to medicines, low adherence represents a challenge to treat chronic conditions, such as COPD [14]. To the best of the authors' knowledge, this study is the first North-African and Maghrebi one raising the issue of pulmonologists' adherence to the 2017-GOLD PTGs. [Supplementary-material supplementary-material-1] ([Supplementary-material supplementary-material-1]) summarizes the adherence variability to some GOLD PTGs observed in the literature. The rational of the study is highlighted in [Supplementary-material supplementary-material-1].

### 4.1. Discussion of Methodology

Discussion related to the sample size and the inclusion criteria is detailed in [Supplementary-material supplementary-material-1]. This study presented some limitations. The first one concerns the adherence concept itself. Although this concept looks clear, its operationalization and its evaluation methods vary among studies and clinical settings [35]. Moreover, the discussion of different but related concepts, including adherence, compliance, concordance, persistence, and some associated terms (e.g., poor, suboptimal, and low), may increase confusion [36]. The aforementioned point was perfectly discussed by López-Campos et al. [12]. In the same line, the factors for analysis of pulmonologists' adherence were collected from the patient factors only, and it was better to collect the pulmonologist factors (e.g., by interviewing them) into data analysis. The second limitation is related to the use of patients' medical records for medical research [37]. In fact, these records are intended for healthcare purposes and not for research. They are therefore not structured to ease the research process [37]. Hence, the data quality of the patients' medical records is a serious obstacle to be taken into account during the study, as poor quality data can lead to biased results [37]. Since incomplete coding of all the patients' data might increase bias in case ascertainments [37], 54 records were excluded from this study. However, the use of medical records for medical research had some advantages such as access to data that are not easily available, large size of data sets, correct identification of COPD, and correct documentation of prescribed therapies [37]. Additionally, the design of the database is set before carrying out the study. So, the study is a post hoc analysis of previously recorded data. The third limitation concerns the lack of patients' record anonymity and the failure to obtain informed consent [37, 38]. In similar studies, obtaining informed consent is expensive and time-consuming. It also leads to bias in terms of responders and nonresponders, and it decreases the generalizability of the research results [38]. Given the lack of patients' contact or intervention, the authors of this study, and after consulting the hospital institutional review board, judged that there was no need for an ethics committee approval. The fourth limitation is related to the study design itself [39]. On the one hand, cohort studies may suffer from selection bias in addition to possible confounding by indication [39]. On the other hand, cohort studies form an appropriate study design to assess associations between multiple exposures and outcomes [39]. They are particularly applicable to study exposures for which randomization is not conceivable for practical or ethical reasons [39]. The last limitation is related to the study one-center design. It was better to include at least one additional center. The benefits of multicenter trials are numerous: rapid recruitment of sufficient numbers of patients and clearer results that are more convincing and more accepting, because the patient sample of multicenter studies is supposed to be representative. However, while Sousse had two university hospitals, only one pulmonology department exists.

### 4.2. Discussion of Results

The evaluation of pulmonologists' adherence to the 2017-GOLD PTGs represents a crucial element in the therapeutic approach for patients with COPD and a current test in hospitals [12]. An effective management requires pulmonologists to correctly adhere to the relevant clinical practice guidelines. This study is the first real-life study in Tunisia and the Maghreb reflecting the clinical practice of pulmonologists through an observational analysis of their adherence to the 2017-GOLD PTGs.

#### 4.2.1. Pulmonologists' Adherence Rates to the 2017-GOLD PTGs

The overall adherence rate of Tunisian pulmonologists to the 2017-GOLD PTGs was 29.7%. This low adherence rate was similar to that observed in a study in Greece [5] (26.3%), but it was lower than others observed in Taiwan [9] (44.9%) and in South Korea [10] (49.6%) ([Supplementary-material supplementary-material-1]). A recent review including 11 studies performed in multiple countries across the globe showed a significant variability in the adherence rates to the GOLD PTGs [40]. For example, the pulmonologists' adherence rates to some other GOLD PTGs varied from 70.1% (Greece) [41] to 18.7% (USA) [7] ([Supplementary-material supplementary-material-1]). The authors of an Italian study including 12 general practitioners reviewed 437 charts and revealed that only 38% of prescriptions were appropriate [42]. In this study, the pulmonologists' adherence rate was dependent on the COPD ABCD groups. The adherence rates were 19.0, 20.6, 8.3, and 39.1%, in groups A, B, C, and D, respectively (Figure 1). However, the above rates were almost lower than the ones reported by Palmiotti et al. [13] (51.0, 54.2, 58.5, and 86.0%, in groups A, B, C, and D, respectively). According to Palmiotti et al. [13], the tendency in Italy is to prescribe the maximum therapy to all patients, irrespective of the degree of severity. Yet, the Tunisian pulmonologists' adherence rates were intermediate compared to those reported in similar studies ([Supplementary-material supplementary-material-1]). The adherence rates varied from 0.2 [43] to 61.5% [16] in group A, from 0.2 [43] to 43.9% [9] in group B, from 0.0 [43] to 81.5% [10] in group C, and from 4.6 [10] to 96.2% [41] in group D. To summarize, COPD patients continue to be treated in the wrong way, signifying that the GOLD PTGs are not being completely utilized [13]. This frequent pattern is not specific to the developing countries, but it is rather an international issue ([Supplementary-material supplementary-material-1]).

In this study, ICS was prescribed in 75.7% of the patients (Table 2). The problem of overprescription of ICS was highlighted in the literature [5, 13, 44, 45]. For example, ICS (alone or with other medicines) was prescribed in 89, 85, 68.4, and 50% of Turkish [44], Greek [45], Greek [5], and Italian [13] COPD patients, respectively. Ten reasons can be advanced to explain this ICS over prescription: (i) ICS is a treatment always available in hospitals; (ii) pulmonologists aim to cover all the stages/groups of COPD; (iii) lack of awareness with regard to the 2017-GOLD PTGs, even among pulmonologists [46]; (iv) repetition of the drugs previously prescribed by another physician without a cautious assessment [13]; (v) presence of wheezing [47]; (vi) high CAT score [47]; (vii) a belief that ICS are more effective [13]; (viii) higher preference for combination devices, including ICS-LABA rather than ICS and LABA in separate devices [13]; and (ix) information received from pharmaceutical companies [13]. In this study, the most inappropriate medical treatments were the associations SABA-ICS in groups A (33.3%) and B (29.9%), SABA-LABA-ICS-Theophylline in group C (33.3%), and SABA-ICS-Theophylline (21.1%) or SABA-ICS (19.9%) in group D (Table 3). Other related studies reported very large frequencies of overprescription (from 85.4% [10] to 2.4% [41]). In this study, prescription of the inappropriate association SABA-ICS is justified by the fact that these two medicines are the only ones available in Tunisian hospitals. In a study in Greece [41], overprescription was identified in all GOLD groups. In COPD GOLD group D, the LABA-ICS-LAMA triple therapy was consistently the most prescribed treatment in many studies [16, 48]. A Taiwanese study explained ICS overprescription in patients belonging to GOLD groups A/B and group D by the presence of wheezing and a high CAT, respectively [47].

The practical management of a complex disease, such as COPD, takes into account the interplay of the clinical severity, the patient's overall well-being, the physicians' experience, and the systems' based resources [49]. Four reasons could be advanced to explain the low adherence rate of Tunisian pulmonologists to the 2017-COPD PTGs: (i) the nonavailability of several treatments in Tunisian public hospitals. In fact, Tunisian hospitals provide SABA, ICS, and some oral bronchodilators. Patients have no access to LABA. (ii) The weak purchasing power of COPD Tunisian patients (i.e., inability to access the treatments); (iii) patients living in rural areas (25.7% in this study) had more difficulties to obtain their treatment; and (iv) different national and cultural attitudes of Tunisian pulmonologists to various classes of drugs [50].

#### 4.2.2. Adherence Barriers to the 2017-GOLD PTGs

In COPD, some specific variables, largely described by López-Campos et al. [12], are associated with PTG adherence. Some of them are physician- (e.g., knowledge), patient- (e.g., understanding), and/or social- (e.g., costs of medication) related factors [17–23]. The Tunisian pulmonologists' adherence to the 2017-GOLD PTGs is influenced by the patients' age, SEL, national health insurance coverage, and GOLD groups (Table 5). Solving the problems related to each of the aforementioned factors will improve the adherence to PTGs [14]. The following sentences will discuss the aforementioned four factors.

The adherence rate increased in older patients (Table 5). This result is in contrast with the one reported by Sharif et al. [49]. They showed that the 2007 COPD PTGs concordant and discordant groups have similar ages [49]. One explanation for this study result could be the coexistence, in elderly patients, of some frequent comorbidities, especially arterial hypertension and diabetes mellitus (Table 2). In fact, most COPD male patients with concurrent cardiovascular disease are more likely to be prescribed bronchodilators [51]. However, since medical treatments of diabetes mellitus and arterial hypertension are totally funded by the national health insurance, Tunisian pulmonologists are sure that COPD treatments will also be reimbursed.

The patient's socioeconomic factors and low-income status have been shown to influence the adherence rate and to be related to the nonuse of medication [14, 52]. Tottenborg et al. [52] examined the impact of both employment and income on the risk of suboptimal adherence to inhaled medication among COPD patients. They showed a higher risk of poor adherence among unemployed (adjusted related risks (aRR): 1.36) and low-income patients (aRR: 1.07). Barriers to adherence related to low income include inconsistent primary healthcare, inability to pay for COPD treatments, and lack of transport [14].

The adherence rate was 2.851 times higher in patients benefiting of the national health insurance coverage when compared to those indigent or without insurance (Table 5). In case of the nonavailability of a national insurance coverage, pulmonologists avoid to prescribe some inhaled drugs (especially LABA and LAMA) since their prices are high and they are sure that patients will not be able to purchase them. The lack of access to resources is also an important barrier. It contributes not only to inappropriate medical treatments but also to under referral of patients to COPD educators and pulmonary rehabilitation [12]. Moreover, it seems that the insurance coverage rules for medications may influence the physicians' prescribing patterns by deviating from the evidence-based guidelines [53].

The adherence rate was almost three times higher in patients classified A/B GOLD groups when compared to those classified C/D (Table 5). This finding is partially in line with those reported in some related studies [6, 9, 13] ([Supplementary-material supplementary-material-1]). First, after adjusting for age, sex, smoking habits, number of exacerbations, control of symptoms, and health service use, Maio et al. [6] showed that group D patients, compared to the ones in group A, are significant protective factors for the lack of prescriptive appropriateness (OR: 0.05). Second, it seems that 8.9% of group D patients are prescribed single bronchodilator therapy by pulmonologists [16]. Pulmonologists also prescribed ICS-deterio triple therapy to 14.3, 27.1, and 57.1% of patients belonging to groups A, B, and C, respectively [16]. Third, Ding et al. [16] reported that patients in groups A and D are more likely to be treated in line with the 2014-GOLD PTGs (61.5 and 77.5%, respectively), compared with 40.1% for group B. Fourth, Palmiotti et al. [13] showed that 49 and 46% of patients in groups A and B were following therapies differently from the 2015-GOLD PTGs, that 41.5% of patients in group C received triple combination therapy, and that 14% of patients in group D did not have a therapy or were following an inappropriate therapy.

In an Italian study, pulmonologists were asked to respond to an online survey aiming to identify the barriers to the 2015-GOLD PTGs and therefore to explain the discrepancy between the guidelines and the clinicians' practices [13]. It appears that (i) 30% of the discrepancy is due to the fact that GOLD recommendations are far from reality; (ii) the “ABCD model” is difficult to regularly use since spirometry is not always possible and it increases the visit duration; (iii) ICS overprescription is due to the information received from the pharmaceutical companies and because ICS are more effective than proven in clinical trials; (iv) inappropriate use of triple therapy is due to the belief that it guarantees better and faster results with regard to the patient's symptoms and it is easier to prescribe than double bronchodilation alone; and (v) physicians and patients underestimate the disease causing 11% of group D patients not to be treated. The following three additional barriers, described in [Supplementary-material supplementary-material-1], were advanced by pulmonologists: disagreement with the guidelines [48], influence of the clinicians' judgment with regard to the validity of some guidelines [49], and lack of perceived benefits [19].

To conclude, the main result of this study was that the adherence rate of North-African pulmonologists to the 2017-COPD PTGs was disappointingly low. Once again, the study results support the established discrepancy between the current real-life practice and the GOLD PTGs in terms of the low reference to COPD management guidelines by pulmonologists, proving that adherence to the GOLD PTGs strategy is far from being optimal.

## Figures and Tables

**Table 1:** Criteria of appropriate and inappropriate treatment according to the 2017-GOLD pharmacological treatment guidelines.

GOLD group	Appropriate treatment		Inappropriate treatment	
	First choice	Second choice	Undertreatment	Overtreatment
A	Bronchodilator	Change the bronchodilator	No bronchodilator	SABA-LAMA SABA-ICS SABA-ICS-Theo SABA-ICS-LABA SABA-ICS-LAMA SABA-ICS-LAMA-LABA SABA-ICS-LABA-Theo SABA-LABA-LAMA

B	LABA or LAMA	LAMA-LABA	Only short-acting bronchodilator	SABA-ICS SABA-ICS-LAMA SABA-ICS-Theo SABA-ICS-LABA SABA-ICS-LABA-Theo SABA-ICS-LABA-LAMA

C	LAMA	LABA-LAMA (if persistence of exacerbations) or LABA-ICS	Only ICS Only LABA Only SABA SABA-ICS SABA-LABA	SABA-ICS-Theo SABA-ICS-Theo-LABA

D	LAMA-LABA	LABA-ICS or LAMA-LABA-ICS (if persistent symptoms or acute exacerbations)	Only ICS Only LABA Only ICS+LAMA Only SABA SABA-LABA SABA-ICS SABA-ICS-Theo SABA-ICS-Theo-LABA SABA-ICS-Theo-LAMA	

GOLD: Global Initiative for Chronic Obstructive Lung Disease; ICS: inhaled corticosteroids; LABA: long-acting *β*-agonist; LAMA: long-acting muscarinic antagonist; SABA: short-acting bronchodilator agonist; Theo: theophylline.

**Table 2:** General characteristics of the chronic obstructive pulmonary disease (COPD) patients (*n* = 296).

Sociodemographic data	Male sex	261 (88.1)
	Age (years)	68 ± 10 [66 to 69] (41-89)
	Younger group	123 (41.5)
	Urban origin	220 (74.3)
Socioeconomic level	High or medium	236 (79.7)
National health insurance coverage	Yes	201 (67.9)
Comorbidity type	Arterial hypertension	62 (20.9)
	Diabetes mellitus	51 (17.2)
	Coronaropathy	37 (12.5)
	Heart rhythm disorder	32 (10.8)
	Heart failure	21 (7.0)
	Dyslipidemia	13 (4.3)
	Arterial occlusion of the lower limbs	8 (2.7)
	Brain stroke	6 (2.0)
	Dysthyroidism	3 (1.0)
Comorbidity	Yes	114 (38.5)
Smoking habits	Current or ex-smoker	264 (89.1)
	Biomass smoke	35 (11.8)
	Mean smoking (pack-years)	61 ± 31 [57 to 65] (5-190)
	Heavy smokers	251 (84.7)
COPD history		
COPD duration	Years	5 ± 5 [4 to 6] (0-40)
	*Duration* < 5 *years*	188 (63.5)
Follow-up before the inclusion in the study	Pulmonologist	193 (65.2)
	No follow-up	60 (20.2)
	General practitioner	43 (14.5)
Mean annual number of exacerbation	Number	3 ± 1 [2 to 3] (0-10)
	Frequent exacerbators	154 (52.1)
Mean number of hospitalizations for acute exacerbation	All live	1.9 ± 1.9 [1.7 to 2.2] (0.0-15.0)
	1 year before the inclusion	0.9 ± 1.0 [0.8 to 1.0] (0.0-13.0)
Functional characteristics and GOLD groups		
Postbronchodilator FEV_1_	(%)	46 ± 21 [44 to 48] (18-118)
Airflow limitation: GOLD	1	27 (9.1)
	2	71 (23.9)
	3	118 (39.8)
	4	81 (27.3)
GOLD groups	A	21 (7.1)
	B	107 (36.1)
	C	12 (4.1)
	D	156 (52.7)
Treatments		
Pharmacological	SABA-ICS	71 (23.9)
	SABA-ICS-Theo	49 (16.5)
	SABA-ICS-Theo-LABA	43 (14.5)
	SABA-LABA-ICS	31 (10.4)
	SABA-LABA-ICS-LAMA	29 (9.7)
	SABA-LABA-LAMA	28 (9.4)
	SABA	24 (8.1)
	SABA-LABA	15 (5.1)
	SABA-LAMA	5 (1.6)
	SABA-LAMA-ICS-Theo	1 (0.3)
Observance of the pharmacological treatments	Yes	251 (84.7)
Nonpharmacological	Smoking cessation among the 264 smokers	147 (55.6)
	Noninvasive ventilation	45 (15.2)
	Long-term oxygen	66 (22.2)
	Influenza vaccination	57 (19.2)
	Pulmonary rehabilitation	0 (0)

Quantitative data were expressed as *mean* ± *SD* [95% confidence interval] (min-max). Qualitative data were expressed as number (%). FEV_1_: forced expiratory volume in one second; ICS: inhaled corticosteroid; LABA: long-acting *β*_2_-agonist; LAMA: long-acting muscarinic antagonist; SABA: short-acting *β*_2_-agonist; Theo: theophylline.

**Table 3:** Percentages of COPD patients with appropriate and inappropriate medical treatments (*n* = 296).

Group A (*n* = 21)			Group B (*n* = 107)			Group C (*n* = 12)			Group D (*n* = 156)		
Appropriate		Inappropriate	Appropriate		Inappropriate	Appropriate		Inappropriate	Appropriate		Inappropriate
SABA only	19.0	33.3^a^	SABA-LABA	9.3	29.9^a^	SABA-LABA-ICS	8.3	33.3^e^	SABA-LAMA-LABA-ICS	11.5	21.1^f^
		19.0^b^	SABA-LABA-LAMA	8.4	13.1^f^			25.0^g^	SABA-LABA-ICS	10.9	19.9^a^
		9.5^c^	SABA-LAMA	2.8	11.2^c^			16.7^h^	SABA-LAMA-LABA	9.6	12.2^e^
		9.5^d^			8.4^d^			8.3^a^	SABA-LABA-ICS-Theo	5.8	5.1^g^
		4.8^e^			8.7^g^			8.3^f^	SABA-LAMA	1.3	1.9^h^
		4.8^f^			8.4^e^						
											0.6^i^
Total	19.0	81.0^∗^		20.6	79.4^∗^		8.3	91.7^∗^		39.1	60.9^∗^
*p*		<0.0001			<0.00001			<0.0001			<0.00001

For the abbreviation, see Table 2. Data were percentages. ^∗^*p* < 0.05 (two-sided chi-square): total appropriate vs. total inappropriate for the same GOLD group. ^a^SABA-ICS. ^b^SABA-LABA-LAMA. ^c^SABA-LABA-ICS. ^d^SABA-LABA-LAMA-ICS. ^e^SABA-LABA-ICS-Theo. ^f^SABA-ICS-Theo. ^g^SABA only. ^h^SABA-LABA. ^i^SABA-LAMA-ICS-Theo.

**Table 4:** Simple univariate analysis: influencing factors of the Tunisian pulmonologists' adherence to the 2017-GOLD pharmacological treatment guidelines (*n* = 296).

Factors	Description	Odds ratio (95% confidence interval)	*p*
Sociodemographic data	Sex (female)	2.797 (1.048 to7.469)	0.0400
	Age (years)	0.971 (0.947 to 0.997)	0.028
	Younger group	1.744 (1.054 to 2.885)	0.0303
	Urban origin	1.146 (0.642 to 2.046)	0.6427
Socioeconomic level	High or medium	4.792 (1.977 to 11.611)	0.0005
National health insurance coverage	Yes	4.205 (2.153 to 8.210)	<0.0001
Comorbidity	Yes	1.509 (0.909 to 2.504)	0.1114
Smoking habits	Current or ex-smoker	3.266 (1.110 to 9.611)	0.0316
	Biomass smoke	0.357 (0.133 to 0.954)	0.0400
	Heavy smokers	1.578 (0.744 to 3.347)	0.2344
COPD mean duration	<5 years	0.714 (0.428 to 1.191)	0.1971
Follow-up before the inclusion in the study	Pulmonologist (vs. others)	2.460 (1.379 to 4.386)	0.0023
Frequent exacerbator	Yes	0.495 (0.297 to 0.826)	0.007
Postbronchodilator FEV_1_ (%)		0.997 (0.985 to 1.009)	0.611
GOLD stages	1-2 (vs. 3-4)	0.990 (0.582 to 1.682)	0.9709
GOLD groups	A-B (vs. C-D)	0.435 (0.255 to 0.742)	0.0022
Nonpharmacological treatment	Smoking cessation among the 269 smokers	1.683 (0.987 to 2.870)	0.0556
	Noninvasive ventilation	1.723 (0.893 to 3.325)	0.1043
	Long-term oxygen	1.616 (0.909 to 2.874)	0.1021
	Influenza vaccination	1.810 (0.992 to 3.302)	0.0528

For abbreviations, see Table 2. *p* (probability): logistic regression.

**Table 5:** Multivariate analysis: influencing factors of the Tunisian pulmonologists' adherence to the 2017-GOLD pharmacological treatment guidelines (*n* = 296).

Factors	Description	Adjusted odds ratio (95% confidence interval)	*p*
Age	Years	0.968 (0.941 to 0.996)	0.025
Socioeconomic level	High or medium	2.950 (1.205 to 7.223)	0.018
National health insurance coverage	Yes	2.851 (1.421 to 5.720)	0.003
GOLD groups	A-B (vs. C-D)	3.009 (1.691 to 5.355)	<0.0001

For abbreviations, see Table 2. *p* (probability): logistic regression.

## Data Availability

The data used to support the findings of this study are available from the corresponding author upon request.

## Abbreviations

aRR: Adjusted relative risks
CAT: COPD assessment test
COPD: Chronic obstructive pulmonary disease
FEV_1_: Forced expiratory volume in one second
FVC: Forced vital capacity
GOLD: Global Initiative for Chronic Obstructive Lung Disease
ICS: Inhaled corticosteroids
LABA: Long-acting *β*-agonist
LAMA: Long-acting muscarinic antagonist
mMRC: Modified British Medical Research Council
OR: Odds ratio
PBD: Postbronchodilator
PTGs: Pharmacological treatment guidelines
SABA: Short-acting *β*-agonist
SAMA: Short-acting muscarinic antagonist
SEL: Socioeconomic level
WHO: World Health Organization.

## Supplementary Materials

Supplementary Materials The appendix includes the following sections: population and methods (i.e., sample size calculation, dyspnea evaluation, and description of the 4 GOLD stages), results (i.e., population general characteristics), and discussion (i.e., rational of the study, discussion of methodology, and description of the adherence barriers to the 2017-GOLD PTGs). Moreover, it includes a table summarizing the adherence variability to some GOLD PTGs observed in the literature.
