# Supplementary material for: Adherence of North-African Pulmonologists to the 2017-Global Initiative for Chronic Obstructive Lung Disease (GOLD) Pharmacological Treatment Guidelines (PTGs) of Stable Chronic Obstructive Pulmonary Disease (COPD)
Source: Biomed Res Int. 2020 Feb 28;2020:1031845. doi: 10.1155/2020/1031845 (PMC7066397; doi:10.1155/2020/1031845)
Supplement: Supplementary Materials — The appendix includes the following sections: population and methods (i.e., sample size calculation, dyspnea evaluation, and description of the 4 GOLD stages), results (i.e., population general characteristics), and discussion (i.e., rational of the study, discussion of methodology, and description of the adherence barriers to the 2017-GOLD PTGs). Moreover, it includes a table summarizing the adherence variability to some GOLD PTGs observed in the literature. [file 1031845.f1.docx]

**APPENDIX A**

**Population and methods**

**Sample size**

The study sample size was estimated using the following formula**[1]**: n= (Z_α/2_^²^ p q)/∆^²^, where “n” was the number of needed patients; “Z_α/2_” was the normal deviate for type-I error (Z_α/2_ = 1.96 for 5% level of significance); “p” was the percentage of patients receiving appropriate pharmacologic treatments; “q” was equal to “1 - p” and “∆” was the precision, fixed at 0.05. According to Staphyla et al.**[2]**, only 26.3% (p=.263) of COPD patients were appropriately medicated according to the 2017-GOLD PTGs. Applying the above data to the formula gave a minimal sample size of 298 COPD patients.

**Data collection**

Dyspnea was evaluated according to the mMRC scale, which is a five-point scale based on degrees of various physical activities that precipitate dyspnea with an increasing severity from scores “0” to “5”**[3]**.

**Applied definitions and classifications**

Four GOLD stages, based on the PBD FEV_1,_ were identified: GOLD 1 (mild: FEV_1_ > 80%), 2 (moderate: 50 % < FEV_1_ < 80%), 3 (severe: 30% < FEV_1_ < 50%) and 4 (very severe: FEV_1_ < 30%)**[4]**.

**Results**

**General characteristics**

Among the 350 medical records, 54 were excluded mainly because of missing data. **Table 1** displays the general characteristics of the 296 COPD patients. A male predominance was noted. Most of the patients were heavy current or ex-smokers, initially followed by a pulmonologist for a COPD discovered less than five years, belonging to GOLD stages 2 or 3 and to GOLD groups B or D, treated by SABA-ICS, having an urban origin and holding national health insurance coverage **(Table 1)**. SABA, ICS, LABA, theophylline, and LAMA were prescribed in 100, 75.7, 49.3, 31.4 and 21.3% of the patients, respectively **(Table 1)**.

**Discussion**

**Rational of the study**

Various studies have proved that the GOLD guidelines focusing on pharmacotherapy is linked to an improvement of airway obstruction and to a decrease in healthcare cost**[5-7]**. Therefore, effective management of COPD requires pulmonologists to adhere correctly to the relevant clinical practice guidelines. COPD PTGs are based on studies with good adherence to treatments**[8]**. However, in everyday practice, therapeutic adherence is far from being perfect and a large inequality of adherence rates to the GOLD PTGs was reported worldwide**[2, 5, 8-21]**. In fact, a significant disconnection has been reported between the guidelines and the clinicians’ practices**[5]**. This non-adherence to PTGs could influence the correctness of prescribing treatments in COPD**[5]**. Nowadays, adherence to therapies emerges as a pertinent task in the daily clinical practice**[8]**. Adherence could be defined as “the degree to which a person’s behavior, following a regimen and/or performing lifestyle variations, corresponds to the approved endorsements from a healthcare provider”**[22]**. This definition is related to the extent to which a physician executes the appropriate therapeutics, or to which a patient achieves the prescribed therapeutic regimen and the perseverance of that accurate achievements over time**[8, 22]**. Medicines will not be effective if physicians do not adhere to the international guidelines and if patients do not follow the prescribed treatments**[22]**. So far, in the developed countries, only 50% of patients with chronic conditions adhere to PTGs**[22]**. In the developing countries, such as the North-African ones, when combined with a meagre access to healthcare, a lack of correct diagnosis and a restricted access to medicines, low adherence represents a challenge to treat chronic conditions, such as COPD**[22]**.

**Table 1S (Appendix A)** summarizes the adherence variability to some GOLD PTGs observed in the literature. The rational of the study is highlighted in the **Appendix A**.

| **Table 1S. Pulmonologists adherence rates and barriers to some GOLD guidelines.** | | | | | | | |
| --- | --- | --- | --- | --- | --- | --- | --- |
| **.1^st^ author [Ref]**  **.Publication Yr**  **.Contry(ies)** | **GOLD guidelines** | **COPD:**  **.Number**  **.% males** | **GOLD: ABCD groups/I-IV stages (%)** | **Adherence rate (%)** | | | **Remarks** |
| **.Kim [11]**  **.2019**  .South Korea | .2011 | .1411 .91.8 | **.A**:16.9  **.B:**43.7  **.C**:5.1  **.D:**34.3 | .**A**:8.1  **.B**:23.0  .**C**:2.2  **.D**:28.2  **All: 61.5** | | | .Influencing factors: NR  .Comparison inappropriate vs. appropriate groups: postBD FEV_1_ (L, %), exacerbation history, GOLD groups, mMRC grade, CAT score  .72.7%: over-treatment  .27.3%: under-treatment  .Common type/inappropriate treatment: overtreatment with ICS |
|  | .2017 | .407  .94.5 | **.A**:32.4  **.B:**59.7  **.C**:1.7  **.D:**6.2 | **.A**:10.6  **.B**:32.5  .**C**:81.5  **.D**:4.6  .**All:**49.6 | | | .Influencing factors: NR  .Sex, postBD FEV_1_ (L, %), FEV_1_/FVC, exacerbation history, GOLD groups, CAT score  .85.4%: over-treatment  .14.6%: under-treatment  .Common types of inappropriate treatment: 45.5% (ICS), 37.5% (LABA+LAMA in group A) |
| **.Stafyla [2]**  **.2018**  .Greece | .2016 | .33  .94 | **.A**:42.4  **.B:**33.3  **.C**:6.1  **.D:**18.2 | .**All**:47.4 | | | .Influencing factors: NR  .50%: inappropriately treated with triple inhaled therapy  .68.4%: ICS  .24.2%:most prescribed treatment: LABA+ICS |
|  | .2017 |  | **.A**:48.5  **.B:**36.4  **.C**:0.0  **.D:**15.1 | **.All:**26.3 | | |  |
| **.Hsieh [10]**  **.2018**  **.**Taiwan | 2011 | .1053  .94.5 | **.A:**18.4  **.B:**40.6  **.C**:6.7  **.D:**34.2 | **.A**:45.4  **.B**:43.9  .**C**:53.5  **.D**:90.8  .**All:**60.9 | | | .Major cause of over-treatment: unnecessary ICS  .Main cause of under-treatment: lack of maintenance LABDs  .GOLD 2011: 29.5%: over-treatment  .GOLD 2017: 46.1%: over-treatment |
|  | 2017 |  | **.A:**23.3  **.B:**63.2  **.C:**1.9  **.D:**11.6 | **.A**:41.6  **.B**:37.7  .**C**:55.0  **.D**:89.0  **.All:**44.9 | | |  |
| **.Foda [15]**  **.2017**  **.**USA | .2011 | .878  .99% | **.I:**9.8  **.II:**43.2  **.III:**33.3  **.IV:**13.8 | **.I**:34.9  **.II**:5.0  .**III**:25.0  **.IV**:34.7  **.All:**18.7 | | | .Influencing factors: NR  .14.2%: over-treatement  .44.0%: under-treatement  .23.1%: incorrect treatment |
|  | | | | | | | |
| **Table 1S. Continued.** | | | | | | | |
| **.1^st^ author [Ref]**  **.Publication Yr**  **.Contry(ies)** | **GOLD guidelines** | **COPD:**  **.Number**  **.% males** | **GOLD: ABCD groups or I-IV stages (%)** | **Adherence rate (%)** | | | **Remarks** |
| **.Ding [14]**  **.2017**  .France, Germany, Italy, Spain, UK, USA | .2014 | .1641  .68.1 | **.A**:10.3  **.B:**45.2  **.C**:1.6  **.D:**42.9 | .**A**:61.5  **.B**:40.1  .**C**:< 40  **.D**:77.5  .**All**:NR | | | .Influencing factors: length of patient diagnosis, CAT score, objective GOLD classification by primary care physician or pulmonologist  .Groups A and D: most likely to be treated in line with GOLD recommendations compared with group B  .Patients with a diagnosis within the past 12 months were more likely to be treated according to recommendations |
| **.Chan [12]**  **.2017**  .Hong Kong | .2011 | .450  .92.2 | **.A**:1.1  **.B:**36.4  **.C**:1.8  **.D:**60.7 | **Baseline**  .A:0.2  .B:1.6  .C:0.0  .D:56.4  .All:58.2 | **Month_6_**  .A:0.2  .B:0.2  .C:0.0  .D:47.2  .All:47.7 | **Month_12_**  .A:0.2  .B:1.0  .C:0.0  .D:50.3  .All:51.6 | .Guideline non-adherence: not associated with increased risk of exacerbation after adjustment of confounding variables  .ICS used in combination especially with LABA: high rate of prescription in all COPD stages.  .Over-treatment of ICS (either alone or with LABA): groups A and B  .Under-treatment of LABDs: group B |
| **.Spyratos [21]**  **.2016**  **.**Greece | .2014 | .342  .NR | **.A**:69.0  **.B:**16.1  **.C**:5.8  **.D:**9.1 | .All:45 | | | .Influencing factors: NR  .53.1%: over-treatment (GOLD groups A and B)  .1.9%: under-treatment  .85%: ICS  .15%: BDs exclusively |
| **.Sen [19]**  **.2015**  .Turkey | 2010 | .719  .85.4 | **.I:**2.2  **.II:**33.1  **.III:**48.1  **.IV:**16.5 | **.I**:6.3  **.II**:14.7  .**III**:84.4  **.IV**:83  **.All:**59.5 | | | .Influencing factors: NR  .Over-treatment: stages I (100%) and II (91.1%)  .Under-treatment: stages III ( 3.3%) and IV (10.9%)  .Lack of treatment: stages II (3.8%); III (2.3%) and IV (5.9%).  .Most administered treatment :43.4%: LABA-ICS-LAMA  .89%: ICS(alone or with BDs) |
| **.Gunen [16]**  **.2015**  .Turkey | .2013 | .1610 .85.7 | .**A**:41.1  **.B**:20.8  .**C**:13.2  **.D**:25.0 | .**A**:6.1  **.B**:8.7  .**C**:18.4  **.D**:79.4  .**All:**26.5 | | | .Influencing factors: NR  .62%: LABA-LAMA-ICS  .56.6%: over-treatment  .3.1%: under-treatment |
| **.Maio [17]**  **.2014**  .Italy | .2008 | .526 .71.2 | **.I:**20.2  **.II:**52.5  **.III-IV:**28.3 | .All:35.6 | | | .Protective factors for lack of prescriptive appropriateness (after adjustment for age, sex, smoking habits, number of exacerbations, control of symptoms, health services use):   - Stages III and IV (compared to stage I): OR: 0.09 - Group D (compared to group A): OR 0.05   .GOLD 2008: over-treatment: 52.8%  .GOLD 2011: over-treatment: 25.0% |
|  | .2011 |  | .**A**:22.2  **.B**:9.7  .**C**:22.4  **.D**:45.6 | .All:61.4 | | |  |
| **Table 1S. Continued.** | | | | | | | |
| **.1^st^ author [Ref]**  **.Publication Yr**  **.Contry(ies)** | **GOLD guidelines** | **COPD:**  **.Number**  **.% males** | **GOLD: ABCD groups or I-IV stages (%)** | **Adherence rate (%)** | | | **Remarks** |
| **.Papala (18]**  **.2013**  .Greece | .2010 | .127  .96.1 | **.I:**7.9  **.II:**39.4  **.III:**37.8  **.IV:**15.0 | **.I**:30.0  **.II**:40.0  .**III**:37.5  **.IV**:57.9  **.All:**40.9 | | | .Influencing factors: NR  .2.4%: under-treatment  .57.5%: over-treatment |
|  | .2011 |  | .**A**:23.6  **.B**:7.1  .**C**:26.8  **.D**:42.5 | .**A**:43.3  **.B**:33.3  .**C**:61.7  **.D**:96.2  .**All:**70.1 | | | .Influencing factors: NR  .3.1%: under-treatment  .28.3%: over-treatment |
| **.Sharif [20]**  **.2013**  .USA | .2007 | .450 .53.8 | **.I:**7.1  **.II:**46.7  **.III:**33.3  **.IV:**12.9 | .56.4 | | | . Age, sex, race, disease severity, co-morbidities: not associated with guideline adherence  .Multivariate analysis: patients co-managed by a primary care physician and pulmonologist had a higher likelihood of receiving guideline-concordant treatment than those managed by one or the other (OR: 4.59)  .7.6%: over-treated |
| **.Corrado [13]**  **.2012**  .Italy | .2008 | .4094  .72.4 | **.I:**18.2  **.II:**42.1  **.III:**22.5  **.IV:**17.2 | **.I-II**:17  **.III-IV**:67  **.All:**37.9 | | | .Exacerbations (presence and number): important trigger for over-treatment in stages I and II  .7.2%: under-treatment  .54.9%: over-treatment |
| **BD**: bronchodilator. **CAT:** COPD assessment test. **COPD**: chronic obstructive pulmonary disease. **FVC:** forced vital capacity. **GOLD**: global initiative for chronic obstructive lung disease. **ICS**: inhaled corticosteroid. **LABA**: long acting β-agonist. **LABDs:** long acting bronchodilators. **LAMA**: long acting muscarinic antagonist. **mMRC:** modified medical research council.  **NR**: not-reported. **OR:** Odds Ratio. **PostBD FEV_1_:** post-bronchodilator 1^st^ s forced expiratory volume. **Yr:** Year. | | | | | | | |

**Discussion of methodology**

The calculated sample size in this study (n=296) “seems” to be satisfactory. It was slightly higher than the samples of some similar studies [n=33**[2]**, n=127**[18]** **(Appendix B)**]. However, it was largely lower than the sample size of some other studies **(Appendix B)**. The calculation of the needed number of patients is fundamental for planning a study protocol. It is a statistically central argument since it ensures adequate power to distinguish statistical significance**[1]**.

In this study, and as for the COPD patients observed in everyday practice in Tunisia, there was a predominance of the male sex and old age. Moreover, as seen in daily practice, most of the patients were heavy or ex-smokers, belonging to GOLD stages 2 or 3 and GOLD groups B or D, and having an urban origin and low SEL**[23-25]**. For example, the mean age of this study patients (68±10 years) was similar to that of COPD patients included in North-African studies [**eg**; 71±6**[24]**, 65±8**[25]**].

**Adherence barriers to the 2017-GOLD PTGs**

The following three additional barriers were advanced by pulmonologists: **i)** disagreement with the guidelines: some pulmonologist prefer to rely on clinical experience since they feel that the guidelines do not adequately account for individual patients’ symptoms or circumstances, including the ability to adhere to the recommended medications**[26]**; **ii)** influence of the clinicians’ judgment with regard to the validity of some guidelines since the majority of randomized clinical trials of COPD management have been sponsored by pharmaceutic companies**[20]**, and **iii)** lack of perceived benefits**[27]**: pulmonologists consider the guidelines as an interference in their freedom in the clinical judgment.

**References**

**1.** Kang M, Ragan BG, Park JH. Issues in outcomes research: an overview of randomization techniques for clinical trials. J Athl Train 2008;43(2):215-221.

**2.** Stafyla E, Kotsiou OS, Deskata K, Gourgoulianis KI. Missed diagnosis and overtreatment of COPD among smoking primary care population in Central Greece: old problems persist. Int J Chron Obstruct Pulmon Dis 2018;13:487-498.

**3.** Fletcher CM, Elmes PC, Fairbairn AS, Wood CH. The significance of respiratory symptoms and the diagnosis of chronic bronchitis in a working population. Br Med J 1959;2(5147):257-266.

**4.** Vogelmeier CF, Criner GJ, Martinez FJ, Anzueto A, Barnes PJ, Bourbeau J, et al. Global strategy for the diagnosis, management, and prevention of chronic obstructive lung disease 2017 report. GOLD executive summary. Am J Respir Crit Care Med 2017;195(5):557-582.

**5.** Palmiotti GA, Lacedonia D, Liotino V, Schino P, Satriano F, Di Napoli PL, et al. Adherence to GOLD guidelines in real-life COPD management in the Puglia region of Italy. Int J Chron Obstruct Pulmon Dis 2018;13:2455-2462.

**6.** Anzueto AR, Kostikas K, Mezzi K, Shen S, Larbig M, Patalano F, et al. Indacaterol/glycopyrronium versus salmeterol/fluticasone in the prevention of clinically important deterioration in COPD: results from the FLAME study. Respir Res 2018;19(1):121.

**7.** Asche CV, Leader S, Plauschinat C, Raparla S, Yan M, Ye X, et al. Adherence to current guidelines for chronic obstructive pulmonary disease (COPD) among patients treated with combination of long-acting bronchodilators or inhaled corticosteroids. Int J Chron Obstruct Pulmon Dis 2012;7:201-209.

**8.** López-Campos JL, Gallego EQ, Hernández LC. Status of and strategies for improving adherence to COPD treatment. Int J Chron Obstruct Pulmon Dis 2019;14:1503-1515.

**9.** Sehl J, O'Doherty J, O'Connor R, O'Sullivan B, O'Regan A. Adherence to COPD management guidelines in general practice? A review of the literature. Ir J Med Sci 2018;187(2):403-407.

**10.** Hsieh MJ, Huang SY, Yang TM, Tao CW, Cheng SL, Lee CH, et al. The impact of 2011 and 2017 global initiative for chronic obstructive pulmonary disease (GOLD) guidelines on allocation and pharmacological management of patients with COPD in Taiwan: Taiwan obstructive lung disease (TOLD) study. Int J Chron Obstruct Pulmon Dis 2018;13:2949-2959.

**11.** Kim TO, Shin HJ, Kim YI, Rhee CK, Lee WY, Lim SY, et al. Adherence to the GOLD guideline in copd management of South Korea: findings from KOCOSS study 2011-2018. Chonnam Med J 2019;55(1):47-53.

**12.** Chan KP, Ko FW, Chan HS, Wong ML, Mok TY, Choo KL, et al. Adherence to a COPD treatment guideline among patients in Hong Kong. Int J Chron Obstruct Pulmon Dis 2017;12:3371-3379.

**13.** Corrado A, Rossi A. How far is real life from COPD therapy guidelines? An Italian observational study. Respir Med 2012;106(7):989-997.

**14.** Ding B, Small M, Holmgren U. A cross-sectional survey of current treatment and symptom burden of patients with COPD consulting for routine care according to GOLD 2014 classifications. Int J Chron Obstruct Pulmon Dis 2017;12:1527-1537.

**15.** Foda HD, Brehm A, Goldsteen K, Edelman NH. Inverse relationship between nonadherence to original GOLD treatment guidelines and exacerbations of COPD. Int J Chron Obstruct Pulmon Dis 2017;12:209-214.

**16.** Gunen H, Yilmaz M, Aktas O, Ergun P, Ortakoylu MG, Demir A, et al. Categorization of COPD patients in Turkey via GOLD 2013 strategy document: ALPHABET study. Int J Chron Obstruct Pulmon Dis 2015;10:2485-2494.

**17.** Maio S, Baldacci S, Martini F, Cerrai S, Sarno G, Borbotti M, et al. COPD management according to old and new GOLD guidelines: an observational study with Italian general practitioners. Curr Med Res Opin 2014;30(6):1033-1042.

**18.** Papala M, Kerenidi N, Gourgoulianis KI. Everyday clinical practice and its relationship to 2010 and 2011 GOLD guideline recommendations for the management of COPD. Prim Care Respir J 2013;22(3):362-364.

**19.** Sen E, Guclu SZ, Kibar I, Bolol U, Yilmaz V, Celik O, et al. Adherence to GOLD guideline treatment recommendations among pulmonologists in Turkey. Int J Chron Obstruct Pulmon Dis 2015;10:2657-2663.

**20.** Sharif R, Cuevas CR, Wang Y, Arora M, Sharma G. Guideline adherence in management of stable chronic obstructive pulmonary disease. Respir Med 2013;107(7):1046-1052.

**21.** Spyratos D, Chloros D, Michalopoulou D, Sichletidis L. Estimating the extent and economic impact of under and overdiagnosis of chronic obstructive pulmonary disease in primary care. Chron Respir Dis 2016;13(3):240-246.

**22.** Adherence to long-term therapies: evidence for action. Geneva, Switzerland: World Health Organization; 2003. Available from: https://www.who.int/chp/knowledge/publications/adherence_report/en/ (last access: February 10^th^ 2020)

**23.** Daldoul H, Denguezli M, Jithoo A, Gnatiuc L, Buist S, Burney P, et al. Prevalence of COPD and tobacco smoking in Tunisia--results from the BOLD study. Int J Environ Res Public Health 2013;10(12):7257-7271.

**24.** Rejeb H, Ben Khelifa M, Ben Abdallah J, Mrad S, Ben Rejeb M, Hayouni A, et al. The Effects of Ramadan-fasting (RF) on inflammatory and hematological indices of stable chronic obstructive pulmonary disease (COPD) male patients: a pilot study. Am J Mens Health 2018;12(6):2089-2103.

**25.** Mosrane Y, Bougrida M, Alloui AS, Martani M, Rouabah L, Bourahli MK, et al. Systemic inflammatory profile of smokers with and without COPD. Rev Pneumol Clin 2017;73(4):188-198.

**26.** Davis KJ, Landis SH, Oh YM, Mannino DM, Han MK, van der Molen T, et al. Continuing to confront COPD international physician survey: physician knowledge and application of COPD management guidelines in 12 countries. Int J Chron Obstruct Pulmon Dis 2015;10:39-55.

**27.** Perez X, Wisnivesky JP, Lurslurchachai L, Kleinman LC, Kronish IM. Barriers to adherence to COPD guidelines among primary care providers. Respir Med 2012;106(3):374-381.
